# The Metric System

**Published:** 1881-05

**Authors:** 


### THE METRIC SYSTEM.

A correspondent—one of many—writes us "I would most earnestly request that you *always* give the weight and measures in the old system (put the metric equivalents in brackets if you choose). I think after this year I will subscribe to such journals only as give the old system."

Our own views on this subject are well known; we have not believed the metric system adapted to the medical practice of this country; but we do hold that every physician should be sufficiently familiar with it to be able readily to convert the one into the other method of measurement.

